# Bioeutectic^®^ Ceramics for Biomedical Application Obtained by Laser Floating Zone Method. *In vivo* Evaluation

**DOI:** 10.3390/ma7042395

**Published:** 2014-03-25

**Authors:** Piedad N. De Aza, Jose I. Peña, Zofia B. Luklinska, Luis Meseguer-Olmo

**Affiliations:** 1 Instituto de Bioingenieria, Universidad Miguel Hernandez, Avda. Ferrocarril s/n, Elche 03202, Alicante, Spain; 2 Department of Science and Technology of Materials and Fluids, Material Science Institute of Aragon, University of Zaragoza-CSIC, c/Maria de Luna 3, Zaragoza 50018, Spain; E-Mail: jipena@unizar.es; 3 Materials Science Department, School of Engineering and Materials Science, Queen Mary University of London, Mile End Road London E1 4NS, UK; E-Mail: z.luklinska@qmul.ac.uk; 4 Unidad de Bioingeniería ósea, Servicio de Cirugía Ortopédica, Hospital Clínico Universitario Virgen de la Arrixaca, Universidad de Murcia, Murcia 30120, Spain; E-Mail: lmeseguer.doc@gmail.com

**Keywords:** calcium phosphate silicate, osseointegration, implantation, *in vivo* evaluation

## Abstract

In this study, the Bioeutectic^®^ blocks were inserted into the critical size defects of eight rabbits, using both tibiae, and the physical and chemical nature of the remodeled interface between the Bioeutectic^®^ implants and the surrounding bone were performed at four and 15 months. The results showed a new fully mineralized bone growing in direct contact with the implants. The ionic exchange, taking place at the implant interface with the body fluids was essential in the process of the implant integration through a dissolution-precipitation-transformation mechanism. The study found the interface biologically and chemically active over the 15 months implantation period. The osteoblastic cells migrated towards the interface and colonized the surface at the contact areas with the bone. The new developed apatite structure of porous morphology mimics natural bone.

## 1. Introduction

Silicon (Si) containing and/or silicon-substituted calcium phosphates (Si-CaP) materials have attracted considerable interest for orthopaedic, oral and maxillofacial applications because of their biocompatibility and tight bonding to bone, resulting in the growth of healthy tissue directly onto their surface, however many outstanding problems still remain unresolved. The most important is the process of total osseointegration of ceramic implants in the human body [1–5].

When dense bioactive materials are implanted into living body, the interaction between bone tissue and these materials usually starts on their surfaces, with the remaining bulk of the material unchanged, often causing a harmful shear stress [3–5], hence, bioactive materials with interconnected porosity in their structure have better potential in hard tissue prostheses [6–8].

The porous structure of bioactive materials provides a template for fibro-vascular ingrowths, which can result in the deposition of new lamellar bone when followed by osteoblasts’ differentiation [9,10]. Porous implants are highly favorable over non-porous type because of the following [6–10]: (a) porous materials have large surface area resulting in a high tendency to bioresorb, therefore, inducing higher bioactivity rate; (b) interconnected pores can provide a framework for bone growth into the matrix of the implant, and, thus, anchor the prosthesis with the surrounding bone, preventing loosening of implants; (c) interconnected porosities act like an organization of vascular canals, which can ensure the blood and nutrition supply for the bone. To serve all these purposes the minimum pore size required in order to regenerate mineralized bone is generally considered to be in the range of 50–100 μm [6–10].

The applications of porous bioactive materials have been restricted as a result of their low fracture strength and fatigue resistance. A new way of making strong porous ceramics with interconnected porosity has been developed by De Aza *et al*. [11–13]. This is based on designing dense bioactive ceramic materials with the ability to develop *in situ* porous hydroxyapatite-like (HA) structure, once the materials are implanted into living body. This material is composed of two phases, pseudowollastonite (α-CaSiO_3_ = psW) of bioactive properties, and α-tricalcium phosphate (α-TCP) of resorbable properties.

**Figure 1.**
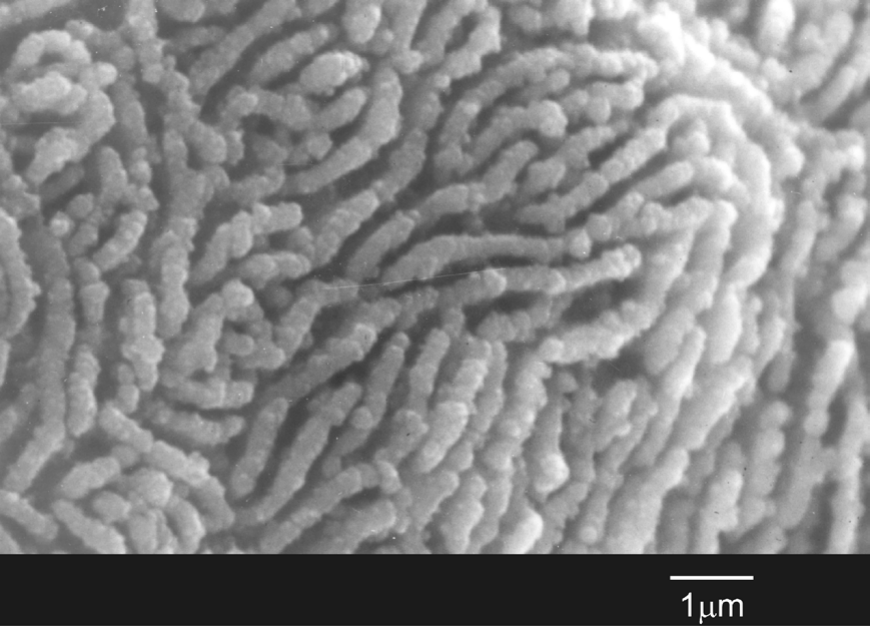
Transformation of the bioeutectic^®^ material after soaking for two weeks in SBF into a structure similar to hydroxyapatite porous bone.

It was found, that during the material exposure to the dynamic simulated body fluid (SBF) for two weeks a complete transformation of the eutectic material takes place, giving rise to a hydroxyapatite porous bone. (Figure 1) [14].

The purpose of the present study was to examine the material osseintegration due to physical and chemical changes taking place at the Bioeutectic^®^ implant-bone interface. It is expected that this material will behave in a similar way as it did in the “*in vitro*” tests, facilitating the osteointegration of the implant. Consequently, the material may present an interesting alternative in biomedical applications.

## 2. Results

**Figure 2.**
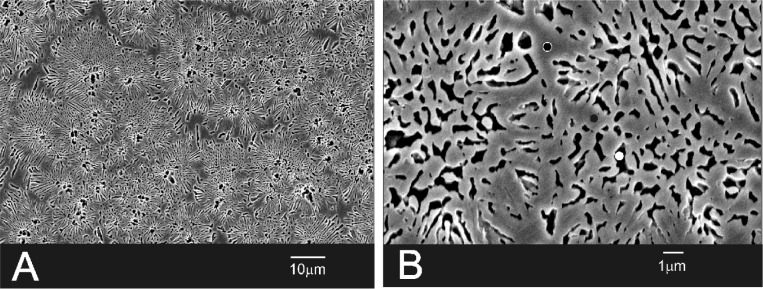
Microstructure of the Bioeutectic^®^ material before implantation chemically etched with dilute acid acetic (○ = TCP; • = psW). (**A**) low magnification; (**B**) high magnification.

SEM morphological observation of the Bioeutectic^®^ material as manufactured showed dense structure constituted of quasi-spherical colonies with an average diameter of ~15 ± 5 μm (Figure 2A). The colonies contained alternating radial lamellae of psW and α-TCP, with an average width of about 1.5 and 0.9 μm, respectively. The morphology of the material corresponded to a fully dense lamellar eutectic structure. SEM-EDX microanalysis showed that the gray phase, as displayed in the images, was composed of psW (•), while the interlocking black phase, that is clearly visible in the micrograph after the chemical etching with dilute acid acetic, was composed of α-TCP (○) (Figure 2B).

All animals survived the 15 months study period without any evidence of inflammation or infection at the implantation sites.

**Figure 3.**
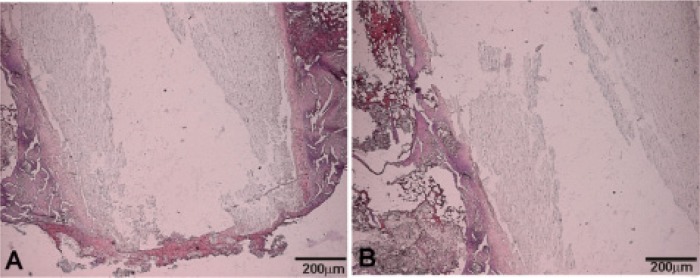
Haematoxylin-eosin stained cross-sections of the implant after four months of implantation. (**A**) Lower part of the implant; (**B**) Middle part of the implant.

After four months of the implantation, the formation of a thin layer of immature bone tissue around the Bioeutectic implant was observed, directly onto the implant surface. After staining, this new layer showed a similar affinity to that of animal bone extracellular matrix surrounding the Bioeutectic implant. Figure 3 presents results of the haematoxylin-eosin stained cross-section of the implant after 4 months of implantation. Figure 3 shows the lower part of the implanted cylinder with the newly-formed bone covering the entire part of the defect. A detail of the implant medullar hematopoyetic tissue interface covered with a new immature bone tissue is presented in Figure 3B. It is important to highlight the absence of any inflammatory cells or formation of any fibrous connective tissue in the vicinity of the implanted material and around the newly formed woven bone, which otherwise would imply intolerance of the bone tissue towards the implant.

**Figure 4.**
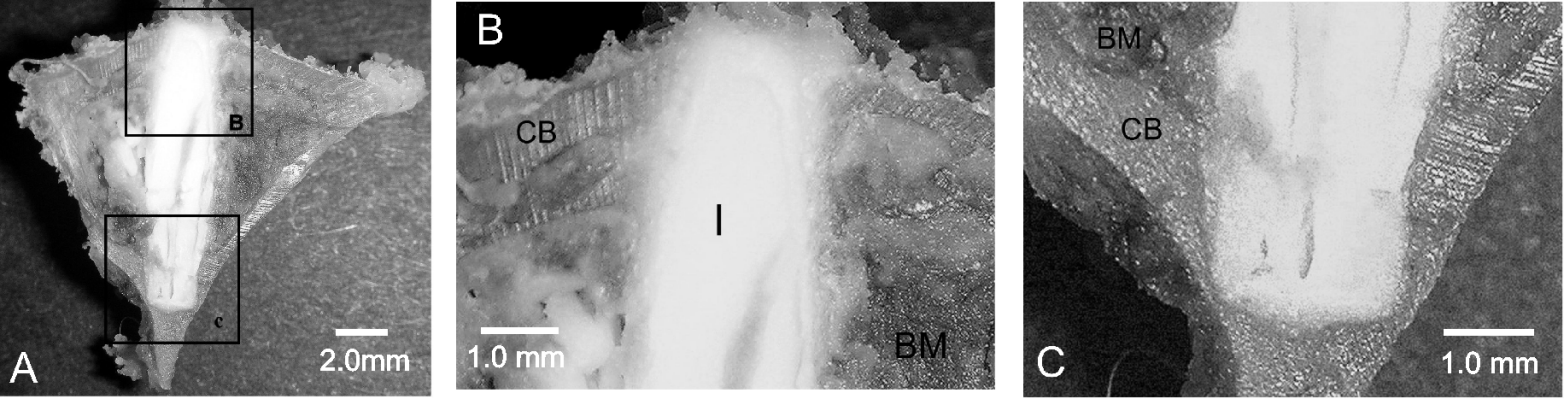
(**A**) Low magnification image of the implant section after 15 months of implantation; (**B**) upper portion of the implant in close contact to the cortical bone; (**C**) close contact between the implant and cortical bone in the lower part of the implant (I: implant, CB: Cortical bone, BM: Bone marrow).

The macroscopic image of the transverse cross- section of the implantation zone after the 15 months periods (Figure 4A) showed that the implanted material (I) strongly adhered to the cortical bone (CB). Figure 4B,C shows the adjacent woven bone formed next to the cortical bone, without interposition of any fibrous tissue, indicating a good osteointegration of the Bioeutectic^®^ implant.

**Figure 5.**
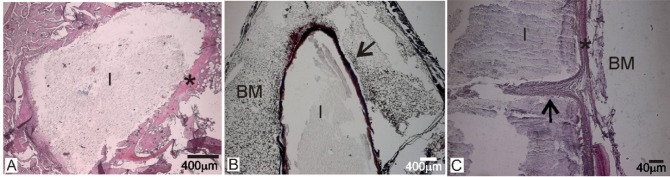
The histological cross-sections of the 15-month implant in bone. (**A**,**C**) stained with haematoxylin-eosin; (**B**) stained with Masson Trichrome. (I: implant, BM: Bone marrow).

After 15 months of the implantation (Figure 5), the general architecture of the implanted material was still visible, although in some areas an irregular surface morphology in contact with the adjacent bone was observed. This was caused by the gradual degradation of the material, due to its progressive reabsorption and substitution by the new mineralized bone tissue, forming trabeculars like natural bone (creeping substitution process). In addition, a high osteoblastic activity and an increase in the quantity of the newly-formed bone tissue in the periphery of the material, between the implant from the hematopoyetic bone marrow, was present. Figure 5A shows the implant (I) located at the centromedular area of the bone surrounded by newly formed bone tissue (*). The transforming implant surface, as a consequence of the reabsorbing process, and a new bone tissue formation around the implant surface (arrow), without interposition of fibrous tissue, is shown in Figure 5B; Figure 5C shows a detail of the implant (I)—hematopoyetic bone marrow interface (*) with the new bone like an osteon (arrow), growing characteristically into the implant (colonization process).

**Figure 6.**
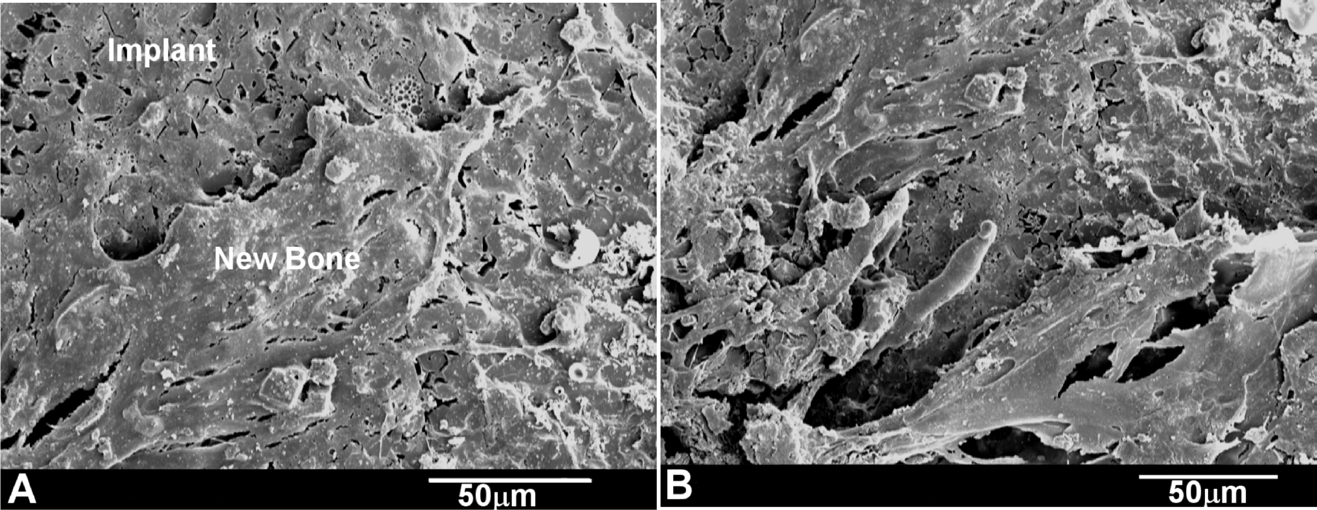
SEM images of the Bioeutectic implant after (**A**) four months of implantation showing the implant surface partially covered by an immature bone tissue; (**B**) at 15 months of implantation showing the whole surface of the implant covered by a newly formed bone tissue.

A strong correlation was found between the SEM implantation results at 4 months and the relevant histological findings. The SEM observation of the 4 month implant showed the new immature bone covering the Bioeutectic^®^ implant, as displayed in Figure 6A. After 15 months of implantation, the whole surface of the ceramic implant was covered by the newly-formed bone tissue (Figure 6B), as the bone deposition process continued.

**Figure 7.**
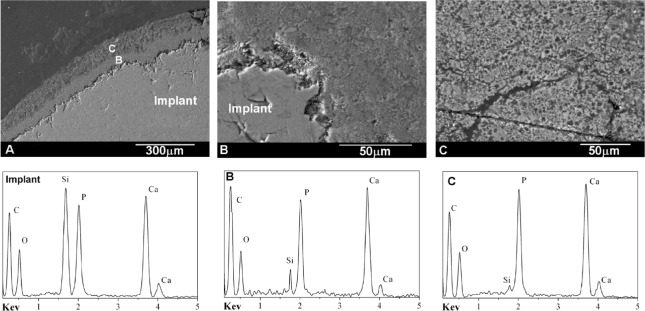
Back-scattered SEM images and EDS analyses of the cross-section of Bioeutectic implant showing the new bone tissue in direct contact with the implant after four months of implantation.

Figure 7 shows the back scattered SEM cross-section images of the interface between the natural bone tissue and the Bioeutectic^®^ implant after four months of implantation. The low magnification image clearly shows the reaction zone at the surface of the ceramic implant composed of two different layers in direct contact with each other. The layers are marked, respectively, as B and C in Figure 7A.

The higher magnification image in Figure 7B shows the interface microstructure between the ceramic implant and the intermediate layer (B) that presents similar morphology of the eutectoid colonies like the morphology of the original implant (Figure 2). Figure 7C shows the newly formed porous bone tissue microstructure in the region (C).

EDS analysis of the implant and the two layers confirmed the chemical compositions of the new bone, the underlayer and the implant substrate. The underlayer (B) was composed of a Ca-P-Si compound, while the new bone over the layer was also composed of the same Ca-P-Si elements but with silicon at relatively low concentration. Quantitative elemental EDS analysis performed at Bioeutectic- bone tissue interface confirmed that the interface remained chemically active over the four-month implantation time. Table 1 lists the Ca/P ratios and Si ion concentration obtained in the bone region at the interface after four months. The microanalysis results indicated that newly grown bone surrounding the implant did not reach the same degree of mineralization within a distance of 150 μm from the interface. The relative Ca/P ratio in the mature area 150 μm away from the interface reach the value of 2.0. In areas closer to the interface, the ratio consistently increased with decreasing distance from the interface to 2.4 at the interface. Also, Si ion concentration decreased with increasing distance from the implant meaning partial dissolution of psW phase.

**Figure 8.**
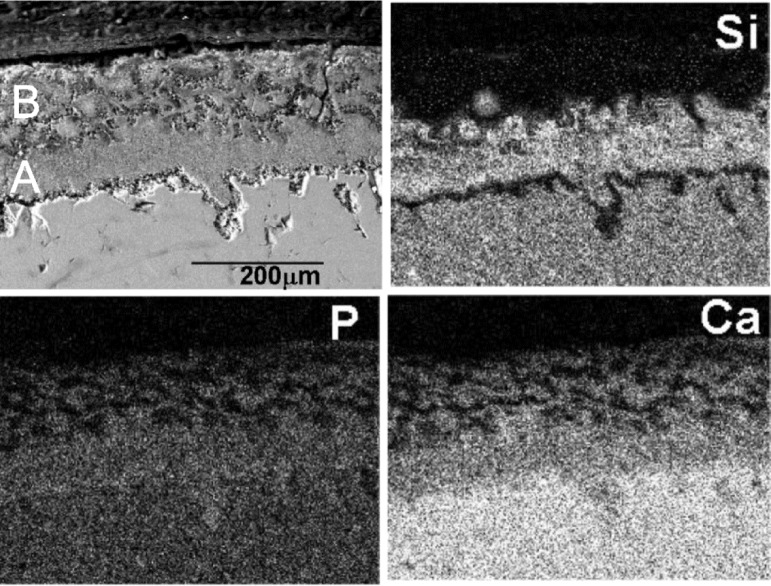
X-ray elemental maps of Si, Ca, P and SEM image of a cross-section of Bioeutectic implant after 15 months of implantation. (**A**,**B**) indicate places where micro-Raman spectra were taken.

Figure 8 shows the SEM microstructure of the polished cross-section of the 15-month implant. The individual X-ray maps of the silicon, calcium and phosphorous distribution are also included. This compositional micro-characterization of the interface showed that there were two chemically separate layers formed on the implant surface. The outer layer contained Ca and P with some traces of Si, while the under layer was rich in Ca, P, and Si. These results agreed with the histological observation described in the study earlier on, confirming formation of the bone phase directly at the implant—natural bone interface.

**Figure 9.**
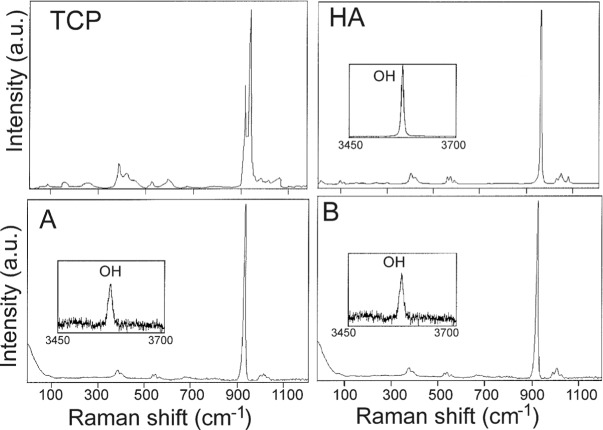
(**A**,**B**) Micro-Raman spectra of the reaction zone after 15 months of implantation. Micro-Raman spectra of Standard TCP and HA is also included.

Figure 9 shows the Micro-Raman spectra collected from of the reaction zone in Figure 8, after 15 months of implantation. Standard TCP and HA are also enclosed for comparative purpose. It is important to emphasize that this technique allowed on differentiation between the original α-TCP phase in the pellets and HA-like reaction products [15–17]. The strong peak observed in Figure 9 at 962 cm^−1^ is assigned to symmetric stretching PO_4_^3−^ modes, and the bands observed in the frequency regions 400–500, 570–625, 1020–1095 cm^−1^ are assigned respectively to ν_2_^−^, ν_4_^−^, and ν_3_^−^ type internal PO_4_^3−^ modes, in agreement with the bibliographic data bases [15–17]. The O–H stretching mode produces an intense Raman peak at 3576 cm^−1^wich is absent from TCP. Consequently, a selective solution of the psW in the medium takes place, leaving a residual phase of HA-like, pseudomorphic with the original α-TCP phase.

**Figure 10.**
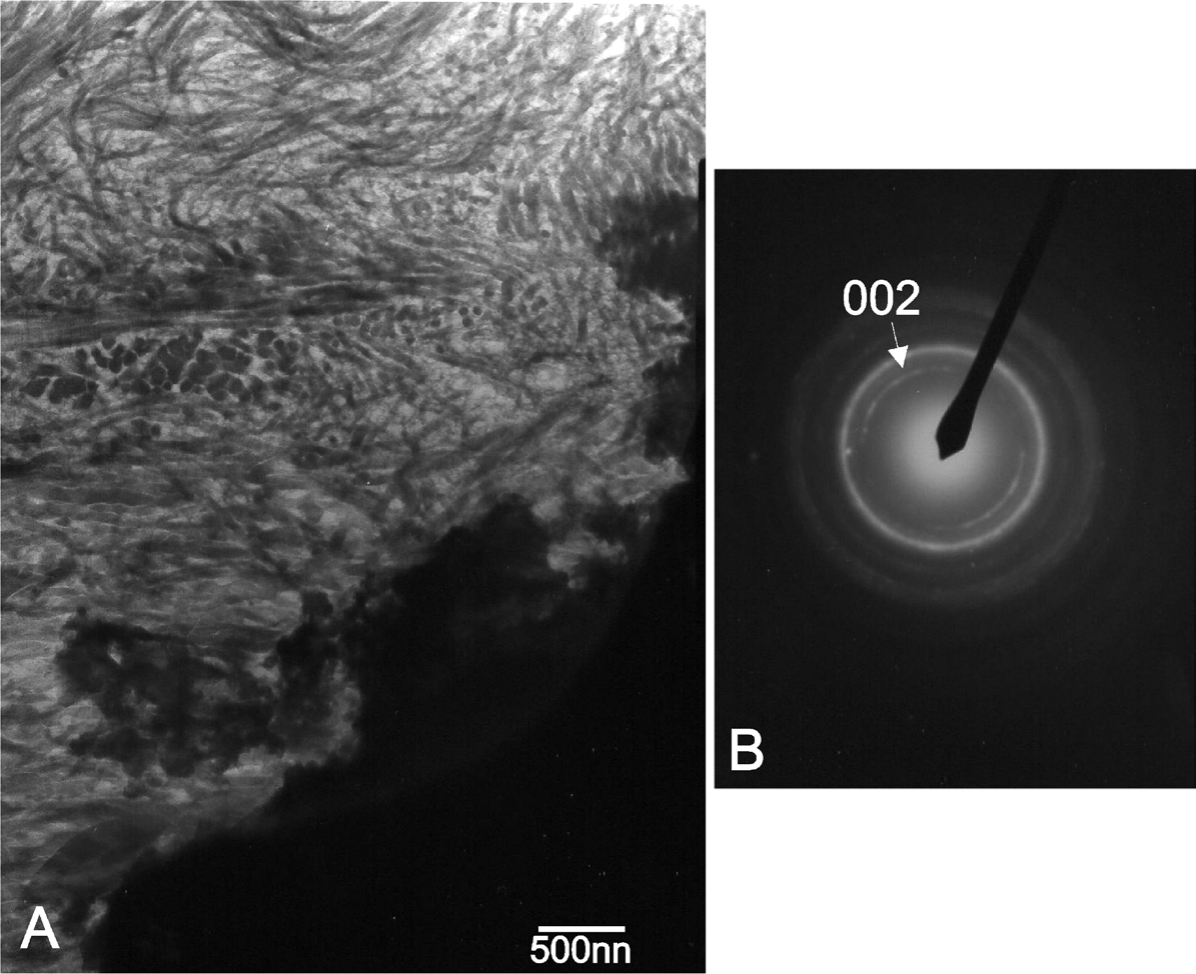
(**A**) Transmission electron micrograph of the stained interface at the 15th month, showing well resolved collagen fibers in the new formed Ca-P phase; (**B**) Equivalent selected area diffraction pattern of the new bone tissue; unstained thin section.

Further identification of the Ca-P phase was carried out by examining thin sections in the transmission electron microscope. This study confirmed that the newly-formed phase mimicked bone from morphological and structural point of view. The bright field image of the stained Ca-P and implant interface is shown in Figure 10A. Collagen fibers with characteristic banding are well resolved in the micrograph. In addition, the selected area diffraction pattern of an unstained thin sections contains the new phase at the interface, Figure 10B, gave rise to typical arcing in the (002) direction due to preferential orientation of the nano-apatite crystals in the matrix of collagen fibers.

## 3. Discussion

The Laser Floating Zone Method enables us to obtain, in the solidification front, very high axial and radial thermal gradients allowing considerable growth speeds compared with other methods or heating sources. On the other hand, the absence of crucibles minimizes the contamination of the samples. This is a very important aspect of the procedure, leading to chemical and structural perfection of the manufactured samples. On the contrary, once of the limitations of the manufacture technique is the diameter size of the cylindrical bioeutectic specimens.

The choice of the New Zealand rabbit model provides a quick and reproducible model for testing bioengineering materials [18,19]. A defect can be defined as critical when it cannot close spontaneously, a situation that is conditioned by specific bone and characteristics of the subject. In the case of New Zealand rabbit model, critical size has been established as 6 mm [19] Due to processing limitations of the method used for the manufactured of the material a housing defect of 3 mm has been used [18]. This defect size facilitated observation of the biomaterial’s activity without interfering in the process of new bone formation but achieving adequate absorption.

A long-term implantation of up to 60 weeks (15 months) was carried out in this investigation, in view of the fact, that the average time required for the integrating of an orthopedic implant in human body is on average three months, while this time is reduced to about four to six weeks in animal models, like rabbits. The experimental observations proved that the implanted material *in vivo* simulation displays strong osseointegration capability. It is also known, that faster bone regeneration at the implanted region, due to the large capacity of bone remodeling in these specimens animals of this weight (3.5 kg equate to an age of 3 months), thus, improving the osseointegration efficacy of the implanted material. In addition, it has taken an intermediate time of four months to see the evolution of the material and not just the final state of the material at 15 months.

The commonly proposed bioactivity mechanism for the Ca-P bioceramics involves initially dissolution of calcium and phosphate ions [1–3] from the material. A subsequent supersaturation of these ions in the vicinity of an implant leads to their precipitation and formation of the biological apatite both heterogeneously on the surface of the implant and also on proteins nearby [20]. Subsequently, this modified surface rapidly absorbs more protein and promotes cell adhesion [21], particularly osteoblasts, which are associated with bone formation and implant bonding [22,23].

In the present study the thickness of the new layer increased significantly during over the first period of four months, from zero to just under 150 μm (Figure 7). From the 4th onwards, the overall reaction rate slowed down. The thickness of the reaction zone at 15 months of implantation reached about 200 μm (±5 μm), of which 120 μm (±5 μm) corresponded to the mineralized new bone tissue and 80 μm (±5 μm) to the under layer of the mature bone tissue (Figure 8). The reaction rate within the first four months was estimated to be about 9 μm per week, compared with 1 μm per week over the next 11 months of implantation.

The already published studies found that an appropriate concentration of silicon in the biomaterial acts as a stimulant for bone matrix integration because of the ion exchange mechanism with the surrounding environment [23–25]. Our extensive literature review on soluble factor effects showed that Si concentrations <19 ppm or Ca concentrations ~250–600 ppm favor osteoblastic differentiation and viability [24–32]. Thus, it appears that there is an optimal concentration range for these soluble elements, within which the cells’ differentiation is stimulated.

This study found a release of Ca, P, and Si ions, which was seen to promote new bone growth; it is possible that high levels of Ca and P stimulate osteogenesis due to their effects on osteoblast gene expression, as described by Lazary *et al.* [32]. In normal calcified bone, the Ca/P molar ratio increases with increasing calcification. For all these elements, the presence of silicon is fundamental, as it effectively promotes mineralization processes. Furthermore, psW-TCP in block form offers biocompatibility, has sufficient mechanical strength and does not produce any adverse inflammatory reactions at the insertion site; the fact that it is absorbable allows its rapid replacement by new bone without causing any reactions to foreign bodies. For this reason, little or no inflammatory reaction was observed; this was also true of the control samples. Its rapid replacement by new bone allows a bone matrix to become established within the material, giving the receiving area physical properties similar to the bone. As we have seen the incorporation of silicon improves the material’s integration and compatibility, and enhances the properties of TCP, which is otherwise reabsorbed too quickly and is less stable.

The reason for the higher Ca/P ratio might be two-fold. The first important factor is continuous Ca ions release from the psW implant. Secondly, the bone could enrich itself with carbonate ions, producing carbonate enriched HA. The mechanism of the formation of the porous new bone can be summarized in the following steps: The psW phase in contact with bone starts to react via an ionic exchange of 2H_3_O^+^ from the medium for one Ca^2+^ from the psW network. This reaction starts at the surface of the material, and progresses deeply into the material, in the confined channels between the α-TCP lamellae as psW is dissolved. Next, the α-TCP lamellae start to react with the Ca^2+^ and OH^−^ ions present in the confined channels. Thus, a pseudomorphic transformation of α-TCP into apatite occurs (Figures 7 and 8). The silicon not involved in the reaction, migrate through the medium away from the interface but the diffusion of the ions across the interface can stop due to the thickness and the structure of the new bone layer. This can be the reason why we found Si in the under layer (Zone A in Figure 8). Next, the bone could enrich itself with carbonate ions, producing carbonate enriched HA of variable CO_3_^2−^ content, and hence giving rise to variable Ca/P ratios, greater than of pure HA, by substituting PO_4_^3−^ for CO_3_^2−^ groups (Table 1).

The indication was that the process of the new bone formation at the interface would continue for as long as the ion exchange mechanism between the implant and the body fluids takes place, assuming that the implant continues to remain in a biologically healthy environment. It is expected that this process will come to the end when the supply of Ca, P, and Si ions from the implant part into the surround terminates. This can occur when the whole implant underwent transformation into the bone phase, and therefore fulfilled its function as the hard tissue substitute material, or if the diffusion of the ions across the interface stopped due to the thickness and the structure of the new bone layer. These results suggested that the Bioeutectic^®^ ceramic remains histologically and morphologically active in the natural hard tissue environment and bonds directly to bone through a Ca-P rich layer containing a trace of Si, mimicking the natural bone in morphology and composition.

In addition, the results from this study indicated that, a solution-mediated effect of the soluble silicate ions on the bone remodeling was important as the silicate ions come from dissolution of the psW phase, hence, may also play a role in accelerating the process of the bone mineralization around the implant. Studies by Carlisle, using electron probe microanalysis, have highlighted that silicon plays an integral role during the bone mineralization processes [33,34]. Silicon is essential for the growth and development of certain biological tissues, such as bone, teeth and some invertebrate skeletons. A recent investigation showed that dietary silicon intake is positively associated with cortical mineral density subject to availability of estrogens in humans [35]. The current study proved that silicon was present in various concentrations in all phases formed on the ceramic surface during the implantation (Figure 7B,C at four month, Figure 8 (elemental mapping of Si) at 15 month, and also Table 1). Although the current paper emphasizes the influence of silicate ions in accelerating the process of the apatite formation on the surface of the implant, it is important to consider both the cell and solution mediated effects of Ca-P-Si ions on the processes of the Bioeutectic osseointegration as it is likely that these two processes occur in parallel at the implant–tissue interface.

The new bone had a similar Ca/P ratio to a natural HA (carbonated hydroxyapatite) and lattice planes of (002) type, related to 0.344 nm spacing of crystalline particle in bone [25,36–38]. For all these elements, the presence of silicon is fundamental, as it effectively promotes mineralization processes. Furthermore, Bioeutetic in block form offers biocompatibility, has sufficient mechanical strength, and does not produce any adverse inflammatory reactions at the insertion site. Its rapid mimic transformation in a new bone allows a bone matrix to become established within the material, giving the receiving area physical properties similar to the bone [39].

Biocompatibility of the material in bone at 15 months post-implantation was determined by the absence of necrosis and inflammation, particularly macrophages and foreign body type of giant cells (FBG). The ability of the material to integrate with the surrounding host bone, otherwise known as osseointegration, was determined by the presence of newly formed bone at the host bone-material interface. The absence of macrophages and fibrous tissue at 15 months post-implantation around the Bioeutectic ceramic and deposition of the new bone at the interface, indicates the biocompatible nature of the material and its good osseointegration properties.

## 4. Experimental Section

### 4.1. Bioeutectic^®^ Preparation

The starting materials were composed of tricalcium phosphate of high-purity (Merck, Darmstadt, Germany) and synthetic pseudowollastonite (the high temperature polymorph of the chain-silicate mineral wollastonite). The psW was synthesized by a solid-state reaction from the stoichiometric mixture of silicon oxide (99.9 wt%, Strem Chemicals Inc, Newburyport, MA, USA) and calcium carbonate (99.5 wt%, Merck, Darmstaadt, Germany). The powders were attrition milled with PSZ-zirconia balls in isopropyl alcohol medium, dried, isostatically pressed at 200 MPa and heated in platinum crucibles at a rate of 5°C/minute up to 1500°C over a four hour period. Next, the psW compound was ground, pressed and reheated again. This procedure was repeated a number of times until the X-ray diffraction analysis (XRD) showed the presence of only the psW phase.

Ceramic precursor rods, 3 mm in diameter and 50–100 mm in length, were prepared from the powder mixture of psW and TCP of the eutectic composition (80 CaSiO_3_ + 20 Ca_3_(PO_4_)_2_ in mol%) by pressureless sintering at 1200°C for 12 h. Eutectic composition and melting point were obtained from the calcium silicate (CS)–Tricalcium phosphate (TCP) phase diagram presented by De Aza *et al*. [40].

The laser floating zone (LFZ) method was used to directionally solidify the eutectic rods. The laser floating zone system consists of a CO_2_ semisealed laser of 600 W (Electronic Engineering, Firenze, Blade600, λ = 10.6 μm), and a home-made growth chamber fitted with gold coated metal mirrors for the beam focusing. The mirror system includes a reflaxicon that transforms the solid beam into a ring, which is deflected by a flat mirror at 45° and focused by a parabolic mirror onto the ceramic rod, undergoing homogeneous heating. The correct optical alignment is obtained with the aid of a red diode laser coaxial providing with the infrared beam. The ceramic rods were attached to the two vertical axis with independent rotation and translation movement. The growth process starts heating the lower end of the precursor. Once a drop is formed a small seed placed in the lower axis is approached until a liquid bridge is established between the precursor and the seed. Then the seed is moved away at the same time as that the precursor is moved to the molten zone, maintaining constant volume of the liquid zone.

The ceramic rods grew at a rate of 20 mm/h at counter rotation of ±15 rpm. Afterwards, the samples were annealed at 650°C for 5 h in order to relieve the stress formed during the quenching process. Small cylindrical bioeutectic specimens, measuring 2.0 ± 0.1 mm in diameter and 10 ± 0.1 mm in length, were cut from the sintered bulk with a diamond saw.

### 4.2. Animal and Surgical Procedure

The experimental protocol was approved by the ethical committee of the University of Murcia following the local and European Regulations.

Eight male adult New Zealand rabbits, weighing between 3.5 and 3.8 kg were divided into two groups of four (*n*_1_ = 4, *n*_2_ = 4) for experiments carried over 4 and 15 months. Both tibiae were used in the implantation procedure. The animals were anaesthetized by an intramuscularly (im) injection of Atropine sulphate, 0.3mg/kg; hydrochlorate of ketamine, 50mg/kg and hydrochloride of clorpromazine, 10 mg/k.

Both legs were shaved and washed with Chlorhexidine^®^, then Betadine^®^ was applied, and, finally, surgical area was covered with sterile drape. After that, an incision 1.2–1.5 cm in length was made in the medial aspect in proximal metaphysial area of each tibia. A bone defects sized 3 mm in diameter were created with spherical surgical drill connected to a micro-motor at low revolutions under continuous saline irrigation to avoid thermal damage to the bone [18]. Cylindrical implants were press fit in the defects to ensure stability. The implants were previously cleaned in sterile PBS solution, dried at 37°C and sterilized with gas plasma. Afterwards, the wounds were closed using meticulous technique (anatomical layers) with continuous sutures (Vicryl™ 3/0 and Vicryl Rapid^®^ 3/0). A single dose of antibiotic therapy (Amoxicilin 0.1 mg/kg/im) was carried out during pre-operative period. Over the next three days after the surgery Tolphenamic acide 0.1 mL/kg/via was administered subcutaneously every 12 h. At the end of 4 and 15 months periods, the animals were euthanized, under sedation (Hydrochlorate of Ketamina, 50 mg/kg/im), with an overdose of thiopental by via intracardiac.

### 4.3. Decalcified and Undecalcified Preparations

The tibia segments containing the implants were excised using a diamond saw 0.5 mm proximal and distal from the implant. Histological analysis was performed on the decalcified tissues. Half of the samples were fixed in 10% neutral formol and decalcified in Hydrochloric-polyvinylpyrrolidone (TBD-1) acid (Termo Shandon, Pittsburg, PA, USA). The decalcified samples were next cleaned and dehydrated in a series of graded ethanol solutions and embedded in paraffin. The regions containing implants were cut into 5 μm thick sections and stained with haematoxylin-eosin and Masson Trichrome for the optical microscopy study using a Leitz Orthoplant FSA microscope (Microscopy-UK, Wallington (Surrey), UK).

The cross-sections of the undecalcified tissues were also examined by the Analytical Scanning Electron Microscopy (SEM). A Hitachi S-3500N Scanning electron microscopy (Hitachi, Ibaraki, Japan) fitted with an energy-dispersive spectrometer (EDS) (Inca-Oxford Instruments, High Wycombe, UK) was used. Secondary electron imaging was performed at 20 keV on carbon coated specimens. These specimens were fixed in 3% glutaraldehyde after soaking in buffer solution overnight, post fixed in 1% osmium tetroxide for two hours at 4°C, washed in 0.1 M cacodilate solution, dehydrated in a series of graded ethanol solutions and finally embedded in hydroxyethylmethacrylate resin. Each undecaldififed block was polished using 6 μm and then 1 μm diamond pastes. In addition, the chemistry of the interfaces was analyzed using X-ray elemental maps. The thickness measurements of the Ca-P layer formed around the implants were performed using back-scattered electron imaging at higher magnification range. Twenty measurements were taken from each sample totaling forty measurements per implantation period.

The samples were also studied by μ-Raman microprobe instrument consisting of a Jobin Ivon T64000 spectrometer (Edison, NJ, USA) fitted with a microscope, which allows a spatial resolution on the sample close to 1 μm. The 488-nm line of an Ar^+^ laser was used as excitation, focused in a spot of ~1 μm in diameter, with an incident power on the sample of ~2 mW.

Morphological and structural identification of the newly-formed Ca-P phase formed at the interface was carried out using Transmission Electron Microscopy (TEM) technique. Bright field images were studied in conjunction with Selected Area Diffraction patterns (SAD). Jeol Jem 2010 microscope (Jeol Ltd., Tokio, Japan) was operated at 200 keV, and 80 cm camera length condition was applied for SAD patterns. Electron transparent thin sections for the study were prepared from the regions of close contact between the implant and new surface product. Reichert ultramicrotome fitted with diamond knife, operated at room temperature, was used for sectioning the material. Morphological study was performed on stained thin sections with saturated solution of uranyl acetate in ethanol and also 1% osmium acid solution in ethanol. Unstained sections were used for structural studies in SAD mode.

## 5. Conclusions

*In vivo* evaluation of the Bioeutectic^®^ ceramic obtained by the laser floating zone method was determined by carried out implantation into tibia of adults New Zealand rabbits. With the limitations of this animal study, it maybe affirmed that Bioeutectic^®^ ceramic is a biocompatible, bioactive, and osteoconductive material. The ceramic material promotes bone regeneration at the site of implantation, does not interfere with normal healing processes and provides an ideal matrix for establishing new bone formation. The Bioeutectic^®^ ceramic implant appeared to act as a physical support where cells with osteoblastic capability were found to migrate and develop processes. These cells colonized the surface of the Bioeutectic^®^ implant in contact with the bone and led to a direct deposition of a new bone, while the implant was undergoing phase transformation into the bone like phase. It is, thus, expected that the Bioeutectic^®^ ceramic could be satisfactorily used for repair or replacement of living bone.

## Figures and Tables

**Table 1.** EDS elemental analysis of the implant-bone interface after four months of study.

EDS elemental analysis	Zone B		Zone C	

	Down	Up	Down	Up
Ca/P	2.4	2.3	2.1	2.0
Si (wt%)	0.59 ± 0.01	0.26 ± 0.01	0.04 ± 0.01	0.01 ± 0.01
